# Global protected area impacts

**DOI:** 10.1098/rspb.2010.1713

**Published:** 2010-11-17

**Authors:** Lucas N. Joppa, Alexander Pfaff

**Affiliations:** 1 Microsoft Research, Computational Ecology and Environmental Sciences, 7 JJ Thomson Avenue, Cambridge CB3 0FB, UK; 2 Duke University, Public Policy and Economics and Environment, Durham, NC 27708, USA

**Keywords:** protected areas, conservation impacts, REDD, land cover, deforestation, matching

## Abstract

Protected areas (PAs) dominate conservation efforts. They will probably play a role in future climate policies too, as global payments may reward local reductions of loss of natural land cover. We estimate the impact of PAs on natural land cover within each of 147 countries by comparing outcomes inside PAs with outcomes outside. We use ‘matching’ (or ‘apples to apples’) for land characteristics to control for the fact that PAs very often are non-randomly distributed across their national landscapes. Protection tends towards land that, if unprotected, is less likely than average to be cleared. For 75 per cent of countries, we find protection does reduce conversion of natural land cover. However, for approximately 80 per cent of countries, our global results also confirm (following smaller-scale studies) that controlling for land characteristics reduces estimated impact by half or more. This shows the importance of controlling for at least a few key land characteristics. Further, we show that impacts vary considerably within a country (i.e. across a landscape): protection achieves less on lands far from roads, far from cities and on steeper slopes. Thus, while planners are, of course, constrained by other conservation priorities and costs, they could target higher impacts to earn more global payments for reduced deforestation.

## 1. Introduction

Protected areas (PAs) have long been the dominant tool for conserving land cover and, thereby, ecosystem services [1–3]. This is likely to continue. For instance, the Convention on Biological Diversity Work Programme on Protected Areas calls for 10 per cent protection of all the world's ecosystems by 2010 (this target will surely be missed [4]).

The evolution of climate policies may also lead to more PAs. To generate tradable credit for avoiding deforestation, nations may choose to lower deforestation below ‘baseline’. The potential to sell such credits provides an incentive to conserve forest by any means, putting a premium on understanding potentially critical roles of PAs in such conservation.

To earn credit requires lowering measured deforestation. Yet PAs tend towards land that, if unprotected, is less likely than average to be cleared [5–7]. Thus, there is reason to feel PAs have not lowered deforestation nearly as much as previously assumed [8–11]. Improving assessment of what parks have done in the past and what current and new PAs can do in the future supports the joint pursuit of both conservation and climate goals, plus their integration with development. This study provides such improved assessments of PAs' impacts upon the maintenance of natural land cover and at a global scale.

Almost all prior assessments of PAs' impacts on land cover do not explicitly address bias in PA location, yielding on average overstatements of PAs' impacts. The source of bias is that PAs are located where clearing threat is relatively low [12]. Without controls for land characteristics relevant for land clearing, the correlation of protection with vegetation can mistakenly suggest causal PA impact [12]. Here, to demonstrate this evaluation issue at a global scale, we mimic a few smaller-scale studies [8–11] by explicitly controlling for characteristics available for all of the 147 countries with over 100 km^2^ of PAs.

The global PA network is composed of national networks that have different histories, including very different suites of motivations for why conservation was enacted. Thus, we analyse every country's PA network in order to provide a large-scale perspective on bias in traditional PA impact estimates while working at a politically relevant resolution. We fully recognize that factors including spatial variation in cost and in biodiversity have shaped and should shape the networks that we observe. Our points still apply widely.

We focus on land-cover outcomes. Despite differences across stakeholders in definitions of ‘PA success’ [13,14], land cover is a useful indicator correlated with species habitat [15] and carbon storage [16]. Land cover is also readily observable [17]. Although carbon policies will probably target forested regions, PAs contain many different vegetation types. As a result, we focus on the broad issue of changes in natural land cover (while acknowledging that the conversion of some natural land cover within a given PA might well be legal and thus not intended to be prevented). We define ‘impact’ as the estimated reduction in natural land-cover conversion resulting from legal land protection.

Our analyses' unique contribution, relative to almost all prior assessments of PA impact, is to demonstrate very broadly the effects on estimated PA impacts of the explicit use of land characteristics to control for variation across a landscape in whether the land that is protected is likely to have had vegetative cover without protection. Limits on global data constrain what we can control, but the influence of a few key control variables for nearly 150 different countries is an explicit demonstration of the global importance of this point.

## 2. Methods

**Figure 1.**
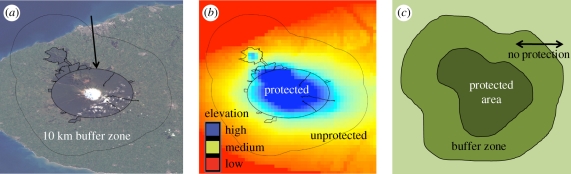
An example of how landscape characteristics influence deforestation. (*a*) Egmont National Park (New Zealand), a common example of non-random location bias of parks. Egmont is a protected volcanic cone containing much of the landscape's remaining forest. (*b*) Sharp elevation gradient at Egmont's boundary with blue representing higher elevation and red lower. Controlling for this elevation is required to accurately estimate Egmont's impacts on retaining forest. (*c*) A caricature of one previous PA impact analysis method. Outcomes such as deforestation would be compared inside the PA boundary with outcomes on the entire unprotected landscape, or within a specified (often 10 km) buffer area around the PA (previous impact method = deforestation rate inside park−deforestation rate outside park, or within 10 km buffer zone).

If PAs were randomly distributed over landscapes, then simply comparing protected with unprotected land could reveal causal impacts of protection [18], since randomness would ensure similarity in land characteristics across these two groups of land parcels. In reality, however, PAs are often located on steep slopes (figure 1) and far from markets [5–7].

We address these differences in protected and unprotected lands' characteristics using ‘matching’. Matching is a treatment or policy evaluation method that can help to reduce the influence of the non-random application of a ‘treatment’ (here legal protection) [18]. For each PA location that is included within such an impact evaluation, matching picks the most similar unprotected sites to best provide ‘apples to apples’ comparisons [9]. The point is that using all the available observed land characteristics to do this matching can greatly improve similarity between treated (protected) and control (unprotected) groups.

For global data, before constructing the most similar apples to apples control groups, we start with a random sample of 5 per cent of each country's PA area (using 1 km^2^ pixel data). We compare this to a random sample, four times as large, drawn from the country's entire unprotected landscape. Our ‘pre-match’ impact estimate for each country subtracts the percentage of natural vegetation in the unprotected sample from that in the PA sample. We do so using: land cover for 2000 [19]; land cover for 2005 [20]; and (despite these 2000 and 2005 datasets not being intended for such comparison) 2000–2005 ‘land-cover change’.

For our ‘post-match’ impact estimate for each country, we are again subtracting the percentage of natural vegetation in the unprotected group from that in the PA group, but now we use a matched subset of the group of unprotected sites. As these characteristics are available, the matching estimates control for land-cover influences of the groups' differences in: elevation; slope; ecoregion; distances to roads and to cities; and agricultural suitability.

Certainly, we do not pretend that these variables fully explain either deforestation pressure or PA location dynamics in any given country. However, they are known to affect profit from agricultural production and thus are often statistically significant predictors of the deforestation rate, for instance. Also, because resistance to PA designation may well rise with land profitability, not surprisingly, they also often correlate with being within a PA. The combination of relevance to PA and land cover makes them useful for our analyses.

The matched unprotected sample is made up by selecting the ‘most similar’ unprotected site for each of our PA sites, with ‘similarity’ defined along these observed dimensions. Specifically, we define ‘most similar’ as ‘shortest distance in land-characteristics space’.

We used ArcGIS 9.3 to harmonize projections, pixel size (to 1 km^2^) and extent. We used Python 2.4 to remove all marine areas and to create individual text files for each variable. We carried out all further analyses in R 2.8.1, using the ‘matching’ package. For each treated location, we chose the single untreated location that was the most similar to it in terms of the multi-variate distance between the locations' vectors of land characteristics (elevation, slope, distances to roads and urban areas, and ecoregion) using the Mahalanobis distance specified by the Abadie & Imbens [18] nearest-neighbour matching approach. Ties between equally similar untreated pixels were broken randomly. When we consider only countries with ‘perfect matching’, significance of covariate imbalance was at the 0.05 level and determined through a bootstrap procedure. For comparison with previous methods, we also calculated a 10 km buffer outside of each PA's boundary. See the electronic supplementary material for further details.

### (a) Land cover—response variable

All data were in raster format. Land-cover data for the year 2000 are from GLC2000 [19] and for 2005 are from GLOBCOVER300 [20]. GLC2000 has 23 classifications of land cover. From those, we reclassified the GLC2000 product into two categories: natural and human-modified. We only included human-modified as those categories identified in the GLC2000 product as such: that is, categories 16 (cultivated and managed areas), 17 (mosaic of cropland with tree cover or other natural vegetation), 18 (mosaics of cropland, with shrubs or grass cover), 19 (bare areas) and 22 (artificial surfaces and associated areas). We classified all other categories as natural. The same process was carried out for the GLOBCOVER300 dataset. The GLOBCOVER300 dataset's legend was meant to be comparable to that of the GLC2000, so we again categorized the land cover into ‘modified’ and ‘natural’. We considered GLOBCOVER300 categories 11 (irrigated croplands), 14 (rainfed croplands), 20 (mosaic cropland 50–70%), 30 (mosaic cropland 20–50%) and 190 (urban areas greater than 50%). Change between the two datasets was calculated after the transformation described above. We recognize this is a noisy estimate of actual land-cover change and thus we do not emphasize those results. However, we do feel it is worth seeing whether the large-scale patterns in the snapshots remain for the change estimate.

### (b) Land characteristics—independent variables

Elevation comes from the Shuttle Radar Topography Mission [21], and we calculated slope in degrees from horizontal. The roads and urban areas used to compute distances are from VMAP0 Roads of the World (all roads in the database were included) [22] and the Global Rural Urban Extent data [23]. While the quality of the VMAP0 data is variable, it is the only freely available dataset to characterize the global road network. We note that urban areas may be stable but some roads may come after PA establishment.

Ecoregions were classified by the World Wide Fund for Nature [24]. Agricultural suitability is from the International Institute for Applied Systems Analysis's Global Agro-Ecological Zones dataset [25]. We use plate 28 of the dataset, which includes climate, soil type, land cover and slope of terrain to measure agricultural suitability, ranking each grid cell from 0 (no constraints) to 9 (severe constraints). These variables are less likely to have shifted after the PA creation.

### (c) Land protection—treatment applied

PAs were from the World Database on Protected Areas (WDPA) [26]. Only countries protecting more than 100 km^2^ of IUCN categories I–VI were included. We considered PAs classified by the IUCN as categories I–VI. In descending order of protection, categories I–IV are for biodiversity protection whereas categories V and VI allow multiple uses. The WDPA contains two types of spatial data on PAs: polygons and points. We only considered those PAs represented by polygons, as the methods required to use the point data can incur serious errors [2]. There was often overlap between PA polygons when converting the PA data to grid format. In each instance, we allowed the most protected IUCN category to determine the category in our dataset. For example, if an overlap occurred between categories I and II, we classified that pixel as category I.

## 3. Results

### (a) Impact estimates

**Figure 2.**
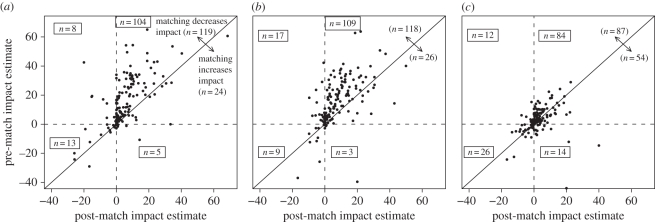
Estimated PA impacts on land cover across 147 countries both before (*y*-axis) and after (*x*-axis) matching. Estimated impact is calculated by subtracting the percentage of natural vegetation of the control sample from the percentage of natural vegetation of the protected sample. Countries above the one-to-one line showed reduced impact estimates as a result of matching. Estimated impacts in the years (*a*) 2000 and (*b*) 2005, and (*c*) the calculated change between 2000 and 2005.

Figure 2 shows pre-match and post-match estimates of PA impacts on natural land cover across the 147 countries with over 100 km^2^ of PA for all IUCN categories of protection (I–VI; below we separate higher and lower protection status). Subfigures convey the pre-match and post-match estimates of the parks' impacts on land cover in the year 2000 (figure 2*a*), land cover in 2005 (figure 2*b*) and 2000–2005 ‘land-cover change’ (figure 2*c*).

Post-match estimates usually indicate positive PA land-cover impacts (i.e. most countries fall in the upper-right quadrants in figure 2*a*–*c*). That is consistent with reduced forest clearing: 75 per cent of countries showed positive land-cover PA impacts for 2000; 76 per cent did for 2005; and 67 per cent showed gains using the noisier estimate of 2000–2005 land-cover change.

Formalizing that these matching estimates usually indicate impacts, a *χ*^2^-test of natural versus converted land cover between treated and control groups frequently finds significance. For the 110 countries with positive estimated land-cover impacts for 2000, approximately 67 per cent of estimates were significantly different from zero (*p*-value >0.05). For the 112 countries with positive impacts for 2005, approximately 76 per cent were significant (*p*-value >0.05). Such tests also help to show the importance of controlling for land characteristics. For 2000 and 2005, respectively, 23 and 26 per cent of the countries with statistically significantly PA impact estimates before matching had insignificant results after matching was applied.

More generally, post-match estimated PA impacts on land cover are significantly lower than are pre-match estimated impacts (i.e. controls for land characteristics really matter). Figure 2*a*–*c* shows a diagonal 1 : 1 line. Controls for land characteristics lower estimated PA impact for countries above the diagonal. Most countries are above the line (2000: 81%; 2005: 80%; ‘change’: 59%). Some fall below but there are more above, and the average for reductions in estimated impact owing to the inclusion of land characteristics (approx. 14% in 2000 land cover) is larger than the average for gains in estimated impact (approx. 6%).

Averaging across all the countries, matching reduced impact estimates by over half of the pre-matching estimate (table 1*a*, ‘catagories I–VI’ shows 2000 is approx. 64%, as the table shows a ratio of the post-match estimated impact to the pre-match; 2005 is approx. 50%). An average that is weighted by PA size produces an even sharper difference (table 1*b*, ‘catagories I–VI’). From this statistical perspective, it appears much of the land-cover impact that pre-match estimates are attributing to the PAs is due to land characteristics and not to the protection itself. That this could be the case even for these few observable factors is quite important.

**Table 1.** Summarized results of global park impacts as averages across all countries. ‘Pre’ and ‘post’ indicate PA impact respectively before and after controlling for landscape characteristics.

	categories I–VI (*n* = 147)^b^	buffer (*n* = 147)^c^	exclude buffer (*n* = 143)^d^	pre-1980 (*n* = 125)^e^	categories I and II (*n* = 110)^f^	categories III and VI (*n* = 110)^g^
(*a*) not weighted^a^						
2000 pre	15.7	13.579	17.343	15.308	17.313	12.732
2000 post	5.715	6.204	7.643	6.185	6.034	6.478
2005 pre	15.299	14.013	16.115	14.767	16.069	11.764
2005 post	7.667	6.348	7.636	8.504	6.29	5.153
change pre	2.78	3.625	2.474	2.735	2.444	1.654
change post	2.85	1.397	1.459	3.055	1.167	0.654
2000 post/pre	0.364	0.457	0.441	0.404	0.349	0.509
2005 post/pre	0.501	0.453	0.474	0.576	0.391	0.438
change post/pre	1.025	0.385	0.59	1.117	0.478	0.396
(*b*) weighted^h^						
2000 pre	14.436	12.192	15.666	12.400	15.047	16.047
2000 post	2.514	2.252	3.233	2.458	3.100	2.639
2005 pre	13.497	11.443	14.475	12.422	14.614	14.660
2005 post	2.250	2.156	2.982	2.537	3.888	2.369
change pre	3.397	3.652	3.526	4.047	4.357	3.307
change post	0.469	0.727	0.743	0.665	1.365	0.607
2000 post/pre	0.174	0.185	0.206	0.198	0.206	0.164
2005 post/pre	0.167	0.188	0.206	0.204	0.266	0.162
change post/pre	0.138	0.199	0.211	0.164	0.313	0.183

^a^A simple average across all country results (i.e. the same weight regardless of treated sample size).

^b^Within a country, treated sample from IUCN category III–VI PAs. Control sample from all unprotected land.

^c^An average weighted on area within the country's network of PAs, generating a more globally representative result.

^d^Within a country, treated sample from all IUCN category I–VI PAs, control sample from all unprotected land.

^e^Same as ‘b’, but control sample from all unprotected land within 10 km of a PA boundary.

^f^Same as ‘b’, but control sample from all unprotected land further than 10 km from a PA boundary.

^g^Within a country, treated sample from IUCN category I–VI PAs created prior to 1980. Control sample from all unprotected land.

^h^Within a country, treated sample from IUCN category I and II PAs. Control sample from all unprotected land.

Ignoring political boundaries to analyse a global sample for the year 2000 is also informative. A random sample of 5 per cent of the world's parks has approximately 94 per cent natural land cover. A comparison with the entire unprotected sample finds 78 per cent natural vegetation, yielding a pre-match impact estimate of 16 per cent. Controlling for land characteristics using matching, however, the post-match impact estimate was only 4 per cent. The results for 2005 are similar.

### (b) Predictable variation in impacts across the landscape

Viewing the matching impact estimates in another way highlights relevance for planning. Post-match estimates for subsamples created by land characteristics reveal that PAs' land-cover impacts vary across a landscape in a given country (see methods in the electronic supplementary material). The PAs within the flattest quartile of a national PA network had a greater impact than PAs on the steepest quartile: across 89 countries, we see higher land-cover impacts for 2000 on flatter land in 54 countries, and higher land-cover impacts for 2005 in 59. Pair-wise comparison of flatter versus steeper shows significantly higher impacts in the flatter regions (one-tailed *t*-test, *p* < 0.001 for 2000 and 2005). The same idea holds for PAs in the closest versus farthest quartiles of the distribution of the distance to urban areas (*n* = 96; one-tailed *t*-test, *p* = 0.011 for 2000 and *p* < 0.001 for 2005).

### (c) Robust findings

One concern when analysing land cover at a single point in time is that for a PA created in 1999, the relationship to 2000 land cover will probably not reflect PA impact on cover. Given the short period for which the PA existed before 2000, it probably reflects the choice to locate the PA where land cover was. To address this, we examine only the parks established before 1980 to check the robustness of our results. In doing so, our sample falls to 125 countries, but our results are similar to those above (table 1*a*,*b*, ‘pre-1980’; electronic supplementary material, figure S3).

Another potential concern is that matching could increase similarity between the groups being compared and yet significant differences could still remain (this generic concern might be of additional interest since we are limited here to globally available data). Thus, we also examine only those countries where we find perfect matching (no significant difference in characteristics) between the protected and the matched unprotected sample. This too reduces our sample; yet results are again similar to table 1*a*,*b* (electronic supplementary material, table S1*a*,*b*).

Finally, as the IUCN protection categories are intended to indicate differing management objectives, it is sensible to replicate analyses for the highest protection status (categories I and II) and separately for PAs of lower status (categories III–VI). These subgroups both show the same pattern as in figure 2 (electronic supplementary material, figures S4 and S5). Average pre-match impact estimates are reduced by at least half after controlling for land characteristics using matching, and PA-size-weighted reductions are even larger (table 1*a*,*b*, ‘categories I–II’ and ‘categories III–VI’). That the reduction in estimated PA impacts from pre- to post-match is greater for category I and II parks than for category III–VI parks matches the expectations from recent results that category I and II PAs are most biased in terms of land characteristics [7].

### (d) Greater similarity than using spatial buffers

Many analysts compare PA outcomes to outcomes in a spatial buffer zone around PAs (figure 1*c*). This assumes, not unreasonably, that drawing from nearby lands generates a control group with the same characteristics. Here, we test the validity of that assumption.

For table 1 (‘buffer’), the pre-match unprotected sample is from lands within 10 km of PA boundaries. If ‘geographical adjacency’ sufficiently equalizes characteristics, then pre- and post-match estimates should be the same. In electronic supplementary material, figure S1 points falling off the 1 : 1 line show this is not the case. Further, while most post-match estimates indicate impact (2000: approx. 70%; 2005: approx. 73%; change: approx. 57%), the critical point is that most (2000: approx. 80%; 2005: approx. 84%; change: approx. 75%) are also lower than the pre-match, *even when the pre-match is drawn from the spatial buffer*. Thus, land characteristics vary between buffers and PAs. The average reduction in the impact estimate is large, again being over half (2000: post-match estimate is approx. 46% or less than half of pre-match; 2005: approx. 45%; change: approx. 39%). Weighting those averages using the PAs' sizes shows even greater reductions (table 1*b*, ‘catagories I–VI’).

As a final robustness check on the importance of controls, we allow that the land cover fate of unprotected lands near a PA could be affected by the PA (e.g. if there is ‘leakage’ or displaced pressure). We redo our analysis, drawing unprotected locations only from further than 10 km from a PA. The results are very similar to those we have already described: most post-match estimates indicate impact; yet they also indicate substantial reduction relative to the pre-match estimates (table 1*a*,*b*, ‘exclude buffer’; electronic supplementary material, figure S2).

## 4. Discussion

Our results suggest that typical analyses have overstated average impacts on land cover, given the fact that PAs tend towards land that is less likely than the average to be cleared. We frequently reject the null that the national PA network had no impact on vegetation. Yet in about 80 per cent of countries, controlling even with our limited land characteristics data lowers the estimated impacts relative to previous methods, such as using spatial buffers. These results suggest some potential benefits from including some areas under high threat. For such areas, matching can easily indicate that typical impact estimates are in fact low.

Such results do not imply criticism of existing PAs' locations or management. Location can be driven by various motivations, and management could be perfect but still have very little land-cover impact if there is very little threat of vegetation loss to be avoided by the protection. Such results do, though, highlight trade-offs in PA location [27], showing that PAs in locations facing little clearing pressure will necessarily prevent little clearing. Naturally, these trade-offs could go either way. For instance, a PA targeting a region of dense and highly valued biodiversity might well be worthwhile even far from roads and cities, as blocking a low threat (i.e. low impact) could provide benefits above all costs. Further, targeting high threats will sometimes be discouraged by correlated high costs.

The second critical feature of these impact estimates is the considerable spatial variation. The PAs closer to roads and cities, and those on flatter land, appear to have higher impacts (i.e. biggest reductions in potential conversion of natural land cover). This variation offers planners an option to target types of locations for higher impacts on the forest (e.g. targeting that could raise earnings if global payments exist for reducing deforestation).

This is important in light of limited resources for such investments. Certainly, one could imagine that almost any location will eventually face clearing pressure at some point in the future. However, resources are insufficient to protect all land (and the price of land reflects the development trade-offs of protecting land that could produce a lot of crops or natural resources). Planners regularly prioritize according to relative benefits and costs, and here we emphasized land-cover-impact benefits of locations under higher pressure. That said, it is likely that these areas are more costly to protect than are low-impact PAs. This further highlights the need for considerable deliberation by conservation planners.

Such results using global data are not intended for policy guidance in any given country. One reason is that while our analysis is geographically and categorically exhaustive (as we examine PAs in multiple management types and 147 different countries), this scope brings limitations. We used a simple dataset with relevant control variables feasible to collect across the entire globe (although we might expect that our corrections would be even stronger with more detailed data for each country). Another reason is that we show that countries differ in the bias of their PA networks towards lands facing lower clearing pressure. Nonetheless, our two critical results (reduced average impact estimates and variation in impact within country) are shown to hold for most of these countries and an even greater share of the existing global PA network. Thus, planners could inform their future protection investment decisions by replicating such analysis in greater local detail. The simplicity yet empirical relevance of the results suggests future value from doing so.
